# Isolation Techniques, Structural Characteristics, and Pharmacological Effects of Phellinus Polysaccharides: A Review

**DOI:** 10.3390/molecules29133047

**Published:** 2024-06-27

**Authors:** Yiming Yin, Xiaolin Shi, Xiaoqing Cai, Fangrui Liu, Wenting Ni, Baohong Li, Xinhuan Wan, Meng Ren

**Affiliations:** 1 College of Pharmacy, Shandong University of Traditional Chinese Medicine, Jinan 250355, China; ymm17862970926@163.com (Y.Y.); sxiaolin411@163.com (X.S.); caixiaoqing0901@163.com (X.C.); fangruiliu840226@163.com (F.L.); 15621892283@163.com (W.N.); 2 College of Pharmacy, Shandong University, Jinan 250100, China; 3 Innovative Institute of Chinse Medicine and Pharmacy, Shandong University of Traditional Chinese Medicine, Jinan 250355, China; sxl13176897681@163.com; 4 College of Physical Education, Shandong University of Traditional Chinese Medicine, Jinan 250355, China

**Keywords:** phellinus polysaccharide, extraction, purification, chemistry, pharmacological activity

## Abstract

Phellinus is a precious perennial medicinal fungus. Its polysaccharides are important bioactive components, and their chemical composition is complex. The polysaccharides are mainly extracted from the fruiting body and mycelium. The yield of the polysaccharides is dependent on the extraction method. They have many pharmacological activities, such as antitumor, immunomodulatory, antioxidant, hypoglycemic, anti-inflammatory, etc. They are also reported to show minor toxic and side effects. Many studies have reported the anticancer activity of Phellinus polysaccharides. This review paper provides a comprehensive examination of the current methodologies for the extraction and purification of Phellinus polysaccharides. Additionally, it delves into the structural characteristics, pharmacological activities, and mechanisms of action of these polysaccharides. The primary aim of this review is to offer a valuable resource for researchers, facilitating further studies on Phellinus polysaccharides and their potential applications.

## 1. Introduction

Phellinus, often referred to as “forest gold”, is a valuable perennial medicinal fungus [1]. It belongs to the following family tree: Basidiomycotina → Hymenomycetes → Aphyllophorales → Hymenochaetaceae → Phellinus. There are mainly three types of Phellinus species: *P. igniarius*, *P. linteus*, and *P. baumii Pilat* [2,3]. Phellinus is parasitic and grows on the trunk of poplar, mulberry, willow, birch, and rhododendron. It is distributed in northeast, north, and northwest China, Sichuan, and Yunnan. Phellinus is widely used in Asian traditional medicine, and its medicinal use was first recorded in Li Shizhen’s Compendium of Materia Medica [4]. The recorded applications of Phellinus in ancient books are as follows: (1) promoting blood circulation, (2) relieving pain, (3) tonifying deficiency, (4) astringent hemostasis, (5) heat clearing, (6) detoxification, etc. [5]. Pharmacological studies have found that Phellinus has antitumor, immune-regulatory, antioxidant, hypoglycemic, and anti-inflammatory activities, etc. [6].

Polysaccharides are among the most significant components of fungi, playing a crucial role in immune regulation and exhibiting notable antitumor activity [7]. Over the past two decades, numerous pharmacologically active metabolites have been isolated and identified from Phellinus. Among these, polysaccharides have been recognized as the most active and significant components. [8]. The polysaccharides of Phellinus exhibit notable pharmacological effects, primarily extracted from the fruiting body and the mycelium. Their chemical composition is complex, and various extraction and purification methods can influence their pharmacological activities. Consequently, this review summarizes the extraction and purification techniques, structural characteristics, and pharmacological activities of polysaccharides obtained from *P. igniarius* and *P. linteus*. We believe this review will serve as a valuable educational resource for researchers, advancing the research and development of Phellinus polysaccharides.

Integrated efforts are essential for the study of antidiabetic drugs, with iminosugars and sugar derivatives playing a key role in this process [9]. Integrated efforts are essential for the study of antidiabetic drugs, with iminosugars and sugar derivatives playing a key role in this process [10]. Studies have shown that sugar-furan and n-butyl-substituted sugar-furan molecules are the most active and highly selective inhibitors [11]. These findings suggest that alternative methods for treating diabetes can be derived from this research, offering valuable insights for the development of new antidiabetic drugs utilizing these polysaccharides or their derivatives in the future.

## 2. Extraction of Phellinus Polysaccharides

Commonly used extraction methods for polysaccharides include solvent extraction, ultrasonic-wave-assisted extraction, microwave-assisted extraction, and enzyme-assisted extraction. The advantages and disadvantages of these methods are summarized in Table 1. The choice of extraction method significantly influences the properties of the polysaccharides. Traditional solvent extraction methods encompass hot water extraction, dilute acid extraction, and alkaline extraction [12]. The solvent extraction method is simple and requires very minimal equipment.

### 2.1. Hot Water Extraction

Hot water extraction is a traditional method for extracting water-soluble polysaccharides from plants, utilizing hot water in a safe and environmentally friendly manner. This method is widely used in current research due to its minimal impact on the chemical structure of the polysaccharides [13]. It is a simple technique that produces high yields of polysaccharides [14]. However, it requires longer extraction times [15].

The yield of polysaccharides is influenced by various factors, including temperature, time, number of cycles, and solid:liquid ratio. Yang et al. [16] optimized the experiment parameters for polysaccharides from *P. igniarius* using an orthogonal experiment, determining the optimal conditions as the temperature at 90 °C, extraction time of 2 h, 2 cycles, and a solid:liquid ratio of 1:50. Dou et al. [17] extracted polysaccharides from pulverized *P. igniarius* fruiting bodies by refluxing for 8 h, repeating the process three times, then combining, concentrating, and freeze-drying the filtrates. Xu et al. [18] optimized the solid:liquid ratio and extraction time at a constant temperature of 100 °C, finding the optimal solid:liquid ratio to be 1:45 with an extraction time of 3.5 h. Li et al. [19] conducted single-factor and orthogonal experiments to optimize the extraction process from *P. igniarius*, reporting the optimum conditions as temperature at 100 °C, extraction time of 2 h, 2 cycles, and a solid:liquid ratio of 1:15. Under these conditions, the reported yield was 6.64%.

### 2.2. Ultrasonic Wave-Assisted Extraction

The ultrasonic extraction method utilizes ultrasonic-assisted solvent extraction, which involves cavitation, vibration, crushing, and stirring. Ultrasonic waves disrupt the cell walls and enhance the penetration of the extraction solvent, allowing it to penetrate the cells of the medicinal materials more effectively. This improves the extraction rate and reduces the extraction time. Additionally, this method avoids the use of high temperatures and prolonged extraction periods, therefore minimizing the impact on the composition of the polysaccharides [20]. Guo et al. [21] investigated the optimum conditions for ultrasonic extraction using an orthogonal experiment, reporting the following optimal parameters: extraction power of 120 W, solid:liquid ratio of 1:40, temperature of 45 °C, and extraction time of 30 min. Under these conditions, the yield of polysaccharides from *P. linteus* mycelium was 107.324 mg/g. Fu et al. [22] reported the optimal conditions for extracting polysaccharides from *P. igniarius* mycelia as follows: extraction power of 210 W, solid:liquid ratio of 1:50, and extraction time of 60 min. The polysaccharide yield under these conditions was 12.78%.

Ultrasonic waves are often combined with other extraction methods, such as enzyme-assisted and microwave-assisted extraction [23,24,25]. Ying et al. [25] employed an ultrasonic-microwave-assisted method to extract polysaccharides from *P. igniarius* fruiting bodies, achieving a reported yield of 10.6%. Additionally, the antitumor and antioxidant activities of these polysaccharides were superior to those obtained using hot water extraction.

### 2.3. Microwave-Assisted Extraction

Microwave-assisted extraction is also a commonly used method. In this technique, low-energy microwaves disrupt the cell wall, releasing intracellular polysaccharides. The advantages of microwave-assisted extraction include high efficiency and selectivity. However, the primary disadvantage is the high cost associated with this method, making it unsuitable for large-scale industrial production [26,27].

Qin et al. [28] optimized the extraction conditions using the single-factor method, determining the optimal parameters as power at 540 W, a solid:liquid ratio of 1:41, and an extraction time of 51 min. Under these conditions, the yield was 4.18%. The influence of various factors was ranked as follows: microwave power > solid:liquid ratio > extraction time. Guo et al. [29] compared microwave-assisted extraction with hot water extraction, reporting a 20.5% increase in yield from *P. igniarius* using microwave-assisted extraction. Shi et al. [27] compared three kinds of extraction methods: hot water, microwave-assisted, and ultrasonic wave-assisted extraction. They found that the yield from microwave-assisted was twice as high as that from hot water extraction and 40% higher than that from ultrasonic wave-assisted extraction. Additionally, the extraction time for microwave-assisted extraction was significantly reduced.

### 2.4. Enzyme-Assisted Extraction

The enzyme-assisted extraction method utilizes enzymes such as cellulase, protease, and pectinase to accelerate the decomposition of plant tissue, therefore dissolving the intracellular polysaccharides in the solvent. This method offers higher extraction efficiency compared to hot water extraction, requiring mild conditions that do not alter the chemical composition of the polysaccharides. Often, enzyme-assisted extraction is combined with ultrasound wave-assisted methods, known as the ultrasonic compound enzyme method, to further enhance extraction efficiency.

Cheng et al. [30] combined ultrasonic waves and complex enzymes to extract the polysaccharides from the fruiting bodies of Phellinus. The material was treated with a combination of cellulase, pectinase, and protease. Using the response surface method to optimize the extraction parameters, the reported optimal conditions were power 360.6 W, solid:liquid ratio 1:32.5, and extraction time 32.7 min. Under these conditions, the yield was 3.31%. Xie et al. [24] employed a sequential extraction method using ultrasonic waves followed by enzyme treatment to extract the polysaccharides from *P. linteus* mycelia. The optimal ultrasonics were extraction time 20 min, solid:liquid ratio 1:25, and power 500 W. The subsequent enzyme treatment conditions were pH 6.5, temperature 50 °C, cellulase 2.5%, pectinase 2.5%, protease 1% and treatment time of 120 min. The polysaccharide yield obtained under these conditions was 6.619%.

### 2.5. Other Methods

Low temperature, low pressure, and alkaline water extraction methods have also been reported [31,32]. The yield and antioxidant activity (TEAC) of polysaccharides obtained from yellow mycelia were 4.41% and 6.33 mM, respectively, under the conditions of an extraction time of 1.8 h, pressure of 0.059 MPa, and liquid-to-material ratio of 52.5 mL/g [31]. During the extraction process, the alkaline solution effectively breaks the hydrogen bonds between cellulose and hemicellulose in the cell wall, allowing insoluble polysaccharides to be released and converted into soluble polysaccharides, significantly improving the extraction rate [32]. Leong et al. [33] examined the structural changes in polysaccharides isolated under acidic and alkaline extraction conditions. They found that, compared to acidic conditions, polysaccharides are more easily hydrolyzed under alkaline extraction conditions. This hydrolysis results in shorter side chains, reduced molecular weight, increased water solubility, and alterations in the molecular spatial arrangement of the extracted polysaccharides.

## 3. Separation and Purification of Phellinus Polysaccharides

The separation of polysaccharides from proteins and pigments can be achieved through deproteinization and decolorization processes, respectively. The polysaccharides obtained from the extraction mentioned typically contain proteins and pigments. Common methods for removing proteins include the Sevag method, the trichloroacetic acid (TCA) method, and the alcohol precipitation method. [34,35,36]. Pigments can be removed using techniques such as carbon decolorization, macroporous resin adsorption, and flocculation [37].

**Figure 1:**
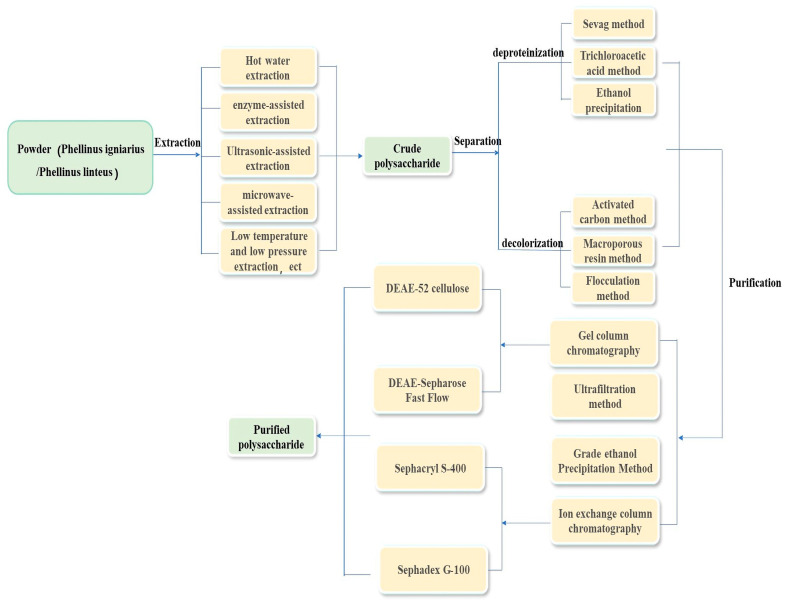
Flow chart of extraction, separation, and purification of Phellinus polysaccharides.

Further purification is required to obtain highly pure homogeneous polysaccharides with stable and functional properties. The main purification techniques include graded alcohol precipitation, ultrafiltration, ion-exchange chromatography, and gel filtration [38,39]. Xie et al. [34] reported that the trichloroacetic acid (TCA) method is more effective than the Sevag method for removing proteins. The optimal conditions for the TCA method are a TCA concentration of 5%, a treatment time of 30 min, and three treatments. Ge et al. [36] compared the effects of alcohol precipitation and flocculation (using chitosan adsorbent) on the loss of polysaccharides from *P. igniarius*, finding that the flocculation method resulted in minimal polysaccharide loss. Xie et al. [40] used DEAE-52 cellulose ion-exchange chromatography and Sephadex G-100 gel filtration chromatography to obtain two homogeneous polysaccharides, Pmae 47000 and Pmae 8700. Ge et al. [41] employed DEAE-Sepharose for purification, developing Fast-Flow ion exchange and Sephacryl S-400 High-Resolution gel filtration methods to isolate pure polysaccharide PBF6. Wu et al. [42] utilized an aqueous two-phase system (ATPS) of choline chloride ([Chol] Cl) and K_2_HPO_4_ to purify the polysaccharides, demonstrating that the antioxidant activity of these polysaccharides was superior to those obtained via alcohol precipitation. The optimal parameters for this method were 68.9% K_2_HPO_4_, 20% [Chol] Cl, a temperature of 21.2 °C, and a treatment time of 30 min. The flow chart of extraction, separation, and purification of Phellinus polysaccharides is shown in Figure 1.

## 4. Chemical Composition and Structure of Phellinus Polysaccharides

### 4.1. Structural Analysis of Phellinus Polysaccharides

The structure of fungal polysaccharides can be categorized into primary, secondary, tertiary, and quaternary levels, with the primary focus of current research being on elucidating the primary structure. The pharmacological activities of polysaccharides are highly dependent on their structures, making structural characterization crucial. Studies aimed at confirming the structure involve determining molecular weight, monosaccharide composition, main chain composition, glycosidic bond type, and more. Commonly used methods for analyzing polysaccharide structure include high-performance liquid chromatography (HPLC) for monosaccharide composition and molecular weight, gas chromatography-mass spectrometry (GC-MS) for determining monosaccharide composition after hydrolysis and derivation, as well as the type of glycosidic bonds [43,44,45,46,47]. Fourier transform infrared spectroscopy (FTIR) is used to identify functional groups and the form of glycoside rings (pyran, furan) [48]. After hydrolysis and derivation, the monosaccharide composition is analyzed using HPLC, GC-MS, and high-performance anion-exchange chromatography (HPAEC) [49]. GC-MS analysis can also determine the type of glycosidic bonds. High-performance liquid and gel chromatography techniques are also commonly used to analyze the molecular weight of polysaccharides. Size exclusion chromatography (SEC) coupled with a multi-angle laser light scattering (MALLS) detector and a differential refractive index (RI) detector is employed to analyze molecular conformation and molecular weight [50]. UV-Vis spectroscopy is used to assess the purity of polysaccharides by scanning over the entire UV range [51]. GC-MS and Nuclear Magnetic Resonance (NMR) can analyze the main and side-chain composition. NMR analyzes main and side-chain compositions, and combined with one-dimensional and two-dimensional spectra, it can infer the linkage and sequence of sugar residues [52]. Additionally, periodate oxidation and Smith degradation can be used to analyze the structure of polysaccharides [17]. These comprehensive techniques ensure a detailed understanding of the structural attributes of polysaccharides, which is essential for correlating their structure with their pharmacological activities.

### 4.2. Chemistry of Phellinus Polysaccharides

The chemical structure of Phellinus polysaccharides is extremely complex, with different polysaccharides being obtained from the same species using various culturing and extraction methods. Phellinus polysaccharides can be categorized into those derived from the fruiting body, mycelium, and extracellular sources (from fermentation broth). Research has primarily focused on the structure of polysaccharides from the fruiting body, particularly *P. igniarius* and *P. linteus*, while the structure of extracellular polysaccharides has received comparatively little attention. The monosaccharide composition of mycelium polysaccharides is more complex than that of the fruiting body, containing higher amounts of fucose and mannose. The molecular weight of polysaccharides from the fruiting body ranges from 10^4^ to 10^6^ Da, whereas polysaccharides from the extracellular matrix have molecular weights greater than 10^6^ Da.

Polysaccharides from the fruiting body are mainly heterogeneous and composed mainly of glucose (50~70%). They also contain mannose, galactose, xylose, arabinose, and rhamnose. Its glycosidic linkages are (1→3), (1→6), or (1→3, 6). Its main chain is →3)-β-D-Glcp-(1→. A study has reported 3 (4)-*O*-methyl-hexose in *P. igniarius* polysaccharides. Ge et al. [53] reported the presence of mannose, glucose, galactose, and 3 (4)-*O*-methyl-hexose in a molar ratio of 0.64:1:1.94: 0.93 PIPF polysaccharide from *P. igniarius*. Kim et al. [54] derived a proteoglycan from the fruiting bodies of *P. linteus* containing mannose, galactose, glucose, arabinose, and xylose. The glycosidic linkages of this proteoglycan were α and β, with β-(1,3)-d-glucose as the backbone. Dou et al. [17] purified two neutral heteropolysaccharides (PL-A (14.2 × 10^3^) and PL-B (22.2 × 10^3^), from fruiting bodies of *P. linteus*. PL-A contains high glucose and low mannose amounts. PL-B is a proteoglycan containing rhamnose. The backbone of both is β-(1→3)—glucan. PL-A contains mannose in the branch and is connected to the backbone via (1→6) linkages. PL-B contains glucose, mannose, rhamnose, and amino acids in the branch that is connected to the backbone via (1→6) and (1→2).

The branches in polysaccharides from mycelium are mainly composed of mannose, galactose, and glucose. Their main chain is mainly composed of (1→3)-D-glucose. Tomoyuki et al. [55] extracted a protein-glucan complex from *P. linteus* mycelium containing 39.3% polysaccharide and connected to glucan via α-(1,3). Kim et al. [56] reported polysaccharide-protein complex (PPC) from the mycelium of *P. linteus* whose molecular weight is 73 KDa and contains mainly D-glucose and D-mannose in the molar ratio of 3:2. Song et al. [57] reported a proteoglycan PLC from *P. linteus* mycelium containing glucuronic acid (6.8%), neutral sugars are mannose (44.2%), galactose (24.1%), glucose (21.1%), arabinose (7.5%) and xylose (3.7%). Wu et al. [58] reported PIP1 polysaccharide (molecular weight, 1.7 × 10^4^ Da) in the fermented mycelium of *P. linteus*, which contains glucose, galactose, and mannose (3.70:4.06:1.00). The glycosidic bond type of the polysaccharide is β. The main chain is (1→3)-glucose and (1→4)-mannose. In addition, Yuan et al. [43] reported high molecular weight (8.12 × 10^5^ Da) heteropolysaccharide PIP-1 from *P. igniarius* fermented mycelium, which is composed of mannose, glucose, and galactose (2.41:87.74:3.86).

The extracellular polysaccharides of Phellinus are obtained from the fermentation broth, and the mycelium polysaccharides typically contain glucose, mannose, and galactose. Lee et al. [59] reported a heteroglycan-protein complex from *P. linteus* with molecular weights ranging from 9.4 × 10^3^ Da to 15 × 10^3^ Da, comprising glucose, galactose, mannose, arabinose, xylose, uronic acid, and amino sugars. Hwang, HJ et al. [47] isolated three components (KCTC6190: Fr-I, Fr-II, and Fr-III) from *P. linteus* with molecular weights of 43.43 × 10^4^ Da, 3.14 × 10^4^ Da, and 1.29 × 10^4^ Da, respectively. These components primarily contain glucose, mannose, and galactose and do not have protein content. Jia et al. [48] reported the separation of a high molecular weight polysaccharide (HHM, 2.84 × 10^6^ Da) and a low molecular weight polysaccharide (HLM, 5.33 × 10^4^ Da) from the fermentation broth. HHM contains glucose, while HLM contains glucose and galactose. The structural characteristics of Phellinus polysaccharides are summarized in Table 2.

## 5. Pharmacological Activities of Phellinus Polysaccharides

### 5.1. Antitumor Activity

**Figure 2:**
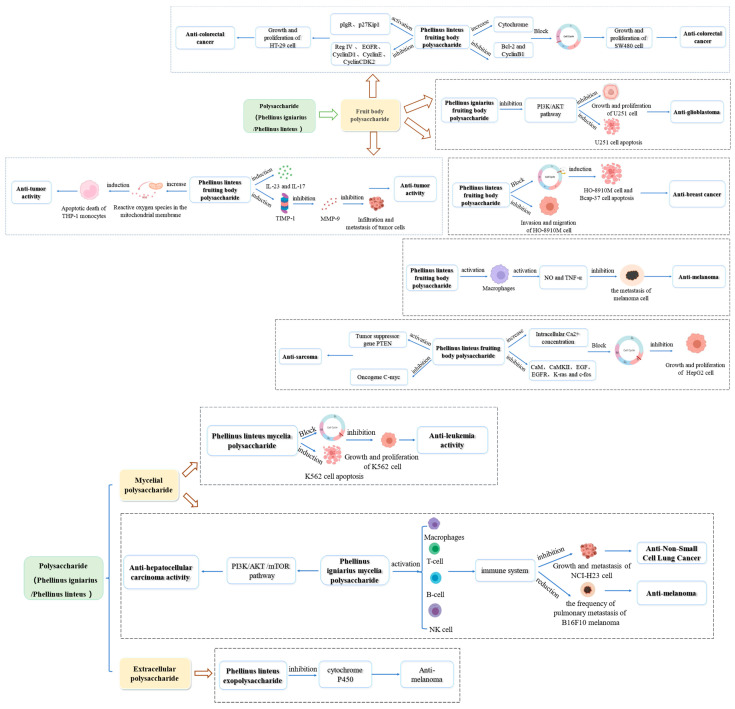
Summary of the antitumor mechanisms of Phellinus Polysaccharides.

Phellinus is known to produce polysaccharides with significant antitumor activity, achieving a tumor inhibition rate of 96.7 % [63]. The primary mechanism of action involves blocking the cell cycle and inducing apoptosis [66,67]. Phellinus polysaccharides have demonstrated antitumor effects on various cancers, including liver, lung, colon, and breast cancers. The specific details of these effects are illustrated in Figure 2.

#### 5.1.1. Antitumor Activity of Polysaccharides from Mycelium

Leukemia is a common malignant tumor of the blood system. It has been reported that intracellular polysaccharides from *P. linteus* significantly inhibit the proliferation of human leukemia cells (K562). The mechanism involves inducing apoptosis and blocking DNA synthesis during the S phase of cell division. However, there are currently no in vivo studies to confirm its activity in a biological system [68].

Hepatocellular carcinoma (HCC) is the most common primary malignant tumor of the liver and ranks second among malignant tumors in terms of its serious impact on people’s health [69]. Currently, the primary therapeutic option for HCC is surgery, as there are no effective drugs available for its treatment. Zhao et al. [70] investigated the antitumor activity of mycelial polysaccharides from *P. igniarius* in a mouse model. The results demonstrated that these polysaccharides inhibit tumor proliferation and enhance the efficacy of cyclophosphamide while reducing its toxicity. The antitumor activity is mediated through the downregulation of the PI3K/AKT/mTOR pathway, vascular endothelial growth factor (VEGF), and inflammatory factors such as TNF-α and IL-6. Wei et al. [71] investigated the antitumor activity of polysaccharides from *P. igniarius* mycelia at three different doses (100, 200, and 400 mg/kg) in hepatoma H22 tumor-bearing mice. The inhibition rates were 26.71%, 34.48%, and 56.65%, respectively. Additionally, the polysaccharides exhibited only mild side effects, and the weight of the mice was not affected. 

Current chemotherapeutic agents often exhibit significant toxic effects and possess only limited efficacy against tumor growth and metastasis [71]. To enhance the efficacy of chemotherapeutic drugs, cytokines, and bacterial products have been utilized as immunochemotherapy agents. Han et al. [72] reported that polysaccharides from *P. linteus* mycelium prolonged the survival rate of B16F10 tumor-bearing mice and reduced the metastasis frequency of melanoma. Additionally, these polysaccharides inhibited tumor growth in the NCI-H23 lung cancer mouse model. It is postulated that *P. linteus* mycelium polysaccharides enhance the activity of T cells, macrophages, natural killer cells, and B cells, therefore inhibiting tumor growth and metastasis through immune system enhancement.

#### 5.1.2. Antitumor Activity of Polysaccharide from Phellinus Fruiting Body

Zhao et al. [73] investigated the antitumor activity of polysaccharides from *P. linteus* fruiting bodies both in vitro and in vivo using a transplanted tumor model in nude mice. The polysaccharides inhibited the growth of breast tumors by 65.37 %. In vitro, they demonstrated inhibitory activity against highly metastatic ovarian cancer (HO-8910M) and breast cancer (Bcap-37) cells, with inhibition rates of 15.53% and 23.81%, respectively. The polysaccharides blocked the G_0_/G_1_ phase of the cell cycle, inhibited the adhesion of tumor cells to the extracellular matrix (ECM), and suppressed cell invasion and migration. Additionally, in vivo experiments showed that crude polysaccharides of Radix Corydalis had a significant inhibitory effect on the growth of Bcap-37 transplanted tumors in nude mice. It was found that *P. linteus* polysaccharides (PL) also directly killed HO-8910PM and Bcap-37 tumor cells cultured in vitro. The comparison of in vivo and in vitro results revealed that the inhibition rate of PL on the growth of transplanted tumors was 65.37%, while the highest inhibition rate on tumor cell proliferation was 23.81%. Furthermore, *P. linteus* polysaccharides inhibited the proliferation of human hepatoma (HepG2) cells. Zhong et al. [74] reported that *P. linteus* polysaccharides mediate their antitumor activity by increasing the intracellular Ca^2+^ concentration. This process down-regulates the expression of several critical genes, including Calmodulin (CaM), Ca^2+^/calmodulin dependent protein kinase II (CaMKII), Epidermal Growth Factor (EGF), epidermal growth factor receptor (EGFR), Kirsten ratsarcoma viral oncogene homolog (K-ras), and cellular oncogene fos (*c-fos*) genes. Additionally, they block the S phase of the cell cycle, contributing to its antitumor effects. Liu et al. [75] studied the in vitro and in vivo antitumor effects of polysaccharides from the *P. linteus* fruiting body using sarcoma S180 cells. The results demonstrated significant antitumor activity both in vitro and in vivo. This activity was attributed to the upregulation of the tumor suppressor gene phosphatase and tensin homolog deleted on chromosome ten (PTEN) and the downregulation of the oncogene C-myc protein. LYU et al. [76] investigated the antitumor activity of polysaccharides from the *P. igniarius* fruiting body at doses of 1.25, 2.5, and 5 g/kg in S180 tumor-bearing mice. The results showed that the polysaccharides exhibited significant antitumor activity and increased the spleen index and thymus index, indicating an enhancement in immune function.

In addition, some studies have found that the antitumor mechanisms of polysaccharides from *P. linteus* fruiting body (CPP) are mediated through the regulation of Tissue Inhibitors of Metalloproteinase 1(TIMP-1), interleukin-23(IL-23), interleukin-17(IL-17), and matrix metallopeptidase 9 (MMP-9) [77]. The tissue inhibitor of metalloproteinase-1 (TIMP-1) complexes with MMP-9 at a 1:1 ratio to inhibit MMP-9 activity [78]. The polysaccharides increased the TIMP-1 expression in RAW264.7 macrophages and mouse liver, contributing to their antitumor effects.

Apoptosis plays a critical role in the suppression of tumor development and can be initiated through external (death receptor-mediated) and internal (mitochondrial-mediated) pathways [79,80]. Griensven et al. [81] investigated the apoptosis of human leukemic mononuclear cells (THP-1) induced by polysaccharides from the fruiting bodies of *P. linteus*. The results demonstrated that these polysaccharides promote apoptosis in THP-1 cells by increasing reactive oxygen species (ROS) in the mitochondrial membrane. This increase in ROS leads to mitochondrial dysfunction, ultimately triggering apoptosis.

Glioblastoma (GBM) is a common primary intracranial tumor and a malignant tumor of the nervous system [82]. The current standard treatment involves surgery combined with radiotherapy and chemotherapy. However, these treatments often lead to immunosuppression, and, over time, chemotherapy can result in drug resistance, affecting the efficacy of the treatment. Wu et al. [83] studied the effects of *P. igniarius* polysaccharides on the proliferation and migration of glioma cells. Their results showed that *P. igniarius* polysaccharides inhibit the proliferation and migration of human glioma U251 cells and promote apoptosis. The underlying mechanism is believed to be related to the inhibition of the PI3K/AKT signaling pathway.

Colorectal cancer is a very common type of cancer. It has been found that *P. linteus* polysaccharide can inhibit the growth and invasion of SW480 colorectal cancer cells both in vivo and in vitro. The mechanism behind this effect is believed to be related to the inhibition of the Wnt/β-catenin signaling pathway [84]. Other studies have found that the inhibitory effect of *P. linteus* polysaccharide on SW480 cell proliferation is achieved by inducing apoptosis, arresting the cell cycle at the G_2_/M phase, decreasing the B-cell lymphoma-2(Bcl-2) and cyclin B1 expression and increasing the cytochrome release [85]. This study lays the foundation for applying *P. linteus* polysaccharides as chemopreventive drugs and immune stimulants. To further explore the mechanism of antitumor activity of *P. linteus* polysaccharide, Li et al. [60] tested the anticancer activity of a purified proteoglycan P1 on HepG2, HT-29, NCI-H460 and MCF-7 cells. It was reported that P1 was effective in these cells, which had a certain degree of antitumor effect, and the best effect was on HT-29 cells. The authors found that P1 can up-regulate the expression of polymeric immunoglobulin receptor (pIgR) and down-regulate the expression of regenerating islet-derived family member 4(RegIV) and EGFR to inhibit cell mitosis and proliferation. Zhong et al. [86] found that P1 can arrest HT29 cells in the S phase of the cell cycle by up-regulating cyclin-dependent kinase inhibitor (p27Kip1) and inhibiting cancer cell proliferation by downregulating cyclin D1 cyclin E and cyclin CDK2. 

Due to the large differences in the polysaccharide structures of *P. igniarius* mycelium and fruiting bodies, their antitumor activities also differ. Studies have found that polysaccharides from *P. igniarius* fruiting bodies have significantly higher antitumor efficacy than polysaccharides from mycelium. Ying et al. [87] compared the in vitro and in vivo antitumor activity of *P. igniarius* mycelial polysaccharide and fruiting body polysaccharide. Cell Counting Kit-8(CCK-8) staining was used in in vitro experiments. In mice, the in vivo activity was tested by administering the polysaccharide intragastrically for 30 days. Both studies showed that polysaccharides from fruit and mycelium had an antitumor effect, which is more for polysaccharides from fruiting bodies. 

Polysaccharides from *P. linteus* fruiting body and mycelium showed different modes of action in inhibiting tumor cell metastasis. Macrophages are essential for maintaining homeostasis and host defense. Kim et al. [88] found that peritoneal macrophages (PM) cultured with mulberry fruiting body polysaccharides (PL) had a dose-dependent killing effect on B16 melanoma cells. At 200 μg/mL, the inhibition rate of PL on cell growth increased by 4 times. Nitric oxide (NO) is the main effector molecule produced by macrophages to destroy tumor cells. Tumor necrosis factor alpha (TNF-α) is an important defense cytokine produced by macrophages against tumor cells. PL has no direct cytotoxicity and can stimulate macrophages to produce NO and TNF-α, thus producing an antitumor metastasis effect. Han et al. [89] found that polysaccharides from mulberry mycelium have two ways to prevent tumor metastasis. On the one hand, polysaccharides from *P. linteus* mycelium can activate host immunity to inhibit tumor metastasis. On the other hand, PL can directly inhibit the adhesion and invasion of tumor cells.

#### 5.1.3. Antitumor Activity of Phellinus Exopolysaccharide

Chemoprevention is the use of chemical or natural substances to prevent cancer development. Chemical prophylaxis mainly acts on the stage of carcinogenesis, among which the conversion of carcinogens to metabolites by cytochrome P450 (CYP) plays an important role. Shon et al. [90] studied the effect of polysaccharides from the fermentation broth of *P. linteus* on the activity of cytochrome P450 isozymes in rat liver microsomes. The results showed that extracellular polysaccharides decreased the activity of Cytochrome P450 (CYP1A1) in a dose-dependent manner and had a strong inhibitory effect on CYP isozymes. It can be concluded that the extracellular polysaccharide of *P. linteus* has much potential to become a chemopreventive agent. However, the in vivo metabolism of extracellular polysaccharides of *P. linteus* remains to be studied.

### 5.2. Antioxidant Activity

**Figure 3:**
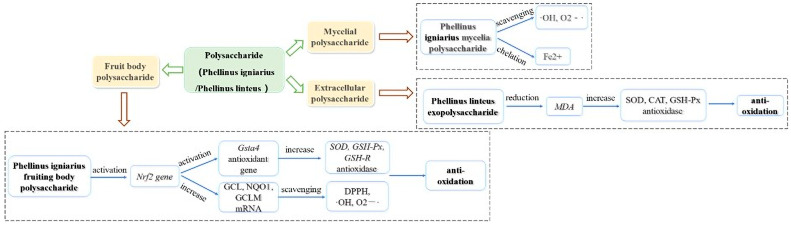
Summary of the antitumor mechanisms of Phellinus Polysaccharides.

Oxidative stress is a negative effect of free radicals in the body, which is an important factor leading to aging and disease [91]. Under normal circumstances, the production and clearance of free radicals in the human body are in a dynamic balance. Once this balance is broken, it will cause body damage and lead to various diseases. Therefore, the research on antioxidant activity mainly focuses on the scavenging of various free radicals. The antioxidant activity of the Phellinus polysaccharide is shown in Figure 3.

#### 5.2.1. Antioxidant Activity of Polysaccharides from Phellinus Mycelium

Yan et al. [61] studied the antioxidant activity of polysaccharides from *P. igniarius* mycelium (IPS) through an in vitro antioxidant model. The results showed that IPS could scavenge ∙OH, O^2−^ and chelated Fe^2+^. Wang et al. [92] analyzed the polysaccharides extracted using different methods (hot water, 1% (NH_4_)_2_C_2_O_4_, and 1.25 M NaOH/0.05% NaBH_4_) by TEAC and Ferric reducing/antioxidant power (FRAP) in vitro. The results showed that the polysaccharides extracted by the three methods showed a certain degree of antioxidant activity. Moreover, the antioxidant activity of these polysaccharides is positively correlated with the activation ability of uronic acid groups.

#### 5.2.2. Antioxidant Activity of Polysaccharide from Phellinus Fruiting Body

*Schistosoma japonicum* can destroy the body’s antioxidant defense mechanism of the body. Zhang et al. [93] established a mouse model infected with *Schistosoma japonicum* to investigate the antioxidant activity of ethanol-extracted polysaccharides (PPI) from *P. igniarius.* The results showed that PPI could significantly improve the body’s antioxidant capacity, increase the content of glutathione (GSH), and restore the activities of antioxidant enzymes such as superoxide dismutase (SOD), glutathione peroxidase (GSH-Px) and glutathione reductase (GSH-R). Its mechanism is related to activating the expression of the nuclear factor erythroid-2-related factor 2(Nrf2) gene and promoting the expression of downstream antioxidant genes such as the glutathione transferase gene, *Gsta4*.

Hu et al. [4] found that polysaccharides from *P. igniarius* fruiting body can scavenge 1,1-diphenyl-2-picryhydrazyl (DPPH), superoxide anion, and hydroxyl radicals. Its mechanism regulates the Nrf2 pathway and increases the mRNA expression of glutamate-cysteine ligase catalytic (GCLC), quinone acceptor oxidoreductase 1(NQO1), and glutamatecysteine ligase, modifier subunit (GCLM).

#### 5.2.3. Antitumor Activity of Phellinus Exopolysaccharide

Yan et al. [65] studied the effects of PL-A11 on antioxidant enzymes in the serum and liver of aging mice. The results showed that PL-A11 could significantly reduce the level of malondialdehyde (MDA) and increase the activities of antioxidant enzymes (SOD, CAT, GSH-Px) in serum and liver. Zhu et al. [94] used a chemical simulation system to compare the antioxidant capacity of intracellular polysaccharides (IPS) and extracellular polysaccharides (EPS). The results showed that the antioxidant capacity of IPS was better than that of EPS. Moreover, the antioxidant capacity of *P. linteus* polysaccharides showed an obvious dose-effect relationship.

### 5.3. Anti-Inflammatory Activity

Inflammation is still one of the most common and serious complications of the disease. Excessive inflammation can lead to a variety of diseases. Few researchers [95] studied the anti-inflammatory activities of intracellular and extracellular polysaccharides of *P. igniarius* using xylene-induced ear inflammation (acute inflammation model) and glaucoma induced by a cotton ball (chronic inflammation model) in mice. The results showed that both intracellular and extracellular polysaccharides had anti-inflammatory effects to a certain extent. Xie et al. [96] showed that *P. linteus* polysaccharide decreased the expression of pro-inflammatory factors (TNF-α, IL-1 β, IL-2, IL-6, IL-12) and increased the expression of anti-inflammatory factors (IL-4, IL-10) in mouse macrophages, Mouse Abelson murine leukemia virus-transformed macrophages (RAW 264.7). NF-κB translocation was significantly inhibited in a dose-dependent manner. Its mechanism involves inhibiting the translocation of NF-κB from the cytoplasm to the nucleus and regulating the balance of anti-inflammatory and pro-inflammatory factors. However, this pathway has not been verified in animal models.

Abdominal infection is often accompanied by intraperitoneal fibrin deposition, leading to abscess and adhesion. Bae et al. [97] showed that *P. linteus* polysaccharides reduced abscess formation and adhesion in a rat model of peritonitis. Its mechanism was related to regulating the fibrinolytic ability of urokinase-type plasminogen activator (uPA) and tissue-type plasminogen activator (tPA) produced by macrophages.

**Figure 4:**
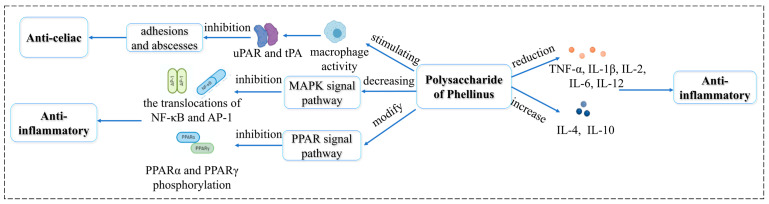
Summary of the Anti-inflammatory mechanisms of Phellinus polysaccharides.

Colitis is a chronic, non-specific inflammation of the colon and rectum, which may cause colon cancer [98]. Hu et al. [99] used lipopolysaccharide-induced inflammation in RAW264.7 cells (in vitro) and sodium dextran sulfate-induced colitis in mice (in vivo) to the effect of polysaccharides from *P. linteus* mycelium. DAI score was used to evaluate the anti-inflammatory efficacy, DAI = (weight loss score + stool score + blood stool score)/3. The results showed that polysaccharides significantly reduced the phenotypic changes and improved the damage to the colon and spleen. Mitogen-activated protein kinase (MAPK) and peroxisome proliferator-activated receptors (PPAR) signaling pathways are closely related to the inflammatory response. It was found that *P. linteus* polysaccharides regulated MAPK and PPAR signaling pathways to inhibit the expression of inflammatory cytokines. The mechanisms involved in the anti-inflammatory activity of Phellinus polysaccharides are shown in Figure 4. 

### 5.4. Immunomodulatory Activity

**Figure 5:**
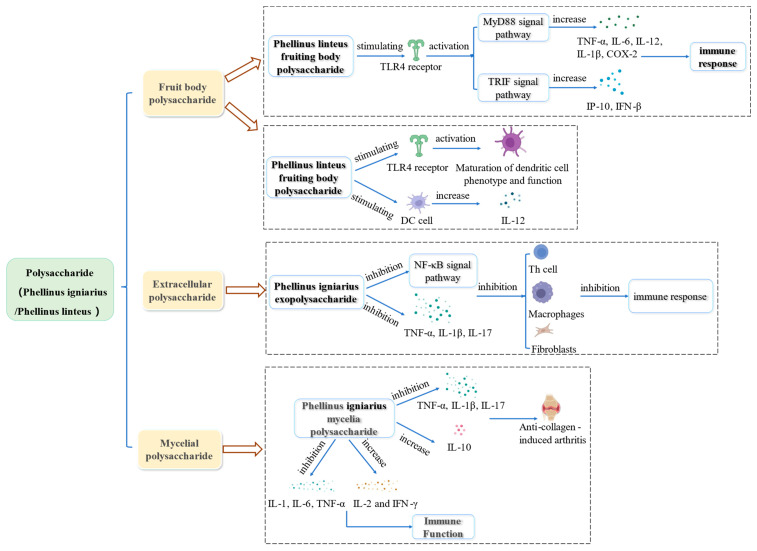
Summary of the immunomodulatory mechanisms of Phellinus polysaccharides.

As an immunomodulator, Phellinus polysaccharide can significantly promote the proliferation of T cells and B cells, increase the production of macrophage secretory factors and NO, and regulate humoral and cellular immunity. The immunomodulatory effect of Phellinus polysaccharides is shown in Figure 5.

#### 5.4.1. Immunomodulatory Activity of Phellinus Exopolysaccharide

Rheumatoid arthritis is a systemic autoimmune disease, and its main lesion is a joint deformity. At present, anti-inflammatory drugs such as glucocorticoids can relieve patients’ pain, but their use is associated with side effects [100]. Li et al. [101] used a collagen-induced arthritis rat model to evaluate extracellular polysaccharides’ efficacy from *P. igniarius*. The results showed a reduction in the degree of toe swelling, improved joint tissue’s pathological condition, and significantly reduced the expression of TNF-α, IL-1β, and IL-17 in serum. Its mechanism is related to the inhibition of the NF-κB signal pathway and the expression of inflammatory cytokines (TNF-α, IL-1β, and IL17). Thus, it reduces the immune response of helper T cells, macrophages, and fibroblasts.

#### 5.4.2. Immunomodulatory Activity of Polysaccharide from Phellinus Mycelium

Li et al. [102] also studied the immunomodulatory effect of polysaccharides from P. *igniarius* mycelium on collagen-induced arthritis in rats. The results showed that the effect of polysaccharides from *P. igniarius* mycelium is the same as that of extracellular polysaccharides. Both inhibited the expression of pro-inflammatory factors (TNF-α, IL-1β, and IL-17). In addition, polysaccharides from *P. igniarius* mycelium can also promote the expression of anti-inflammatory IL-10 and play a therapeutic role in CIA rats.

Wei et al. [71] studied the effect of *P. igniarius* polysaccharides (100, 200, and 400 mg/kg) on the proliferation of T and B lymphocytes in hepatoma H22 tumor-bearing mice. T lymphocytes are mainly responsible for regulating cellular immunity, while B lymphocytes are mainly responsible for regulating humoral immunity. The results showed that polysaccharides promoted the proliferation of T and B lymphocytes in splenocytes, the body’s humoral immunity, and cellular immunity. The authors also studied the effect of liquid-fermented *P. igniarius* mycelium polysaccharide on the immune function of normal mice. It enhanced the carbon clearance in mice, increased the levels of IL-2 and Interferon-γ(IFN-γ), and decreased the levels of IL-1, IL-6, and TNF-α [103]. It can be seen that the mycelium polysaccharide of *P. igniarius* has the effect of an immune enhancer, which can significantly enhance the immune function of the body.

#### 5.4.3. Immunomodulatory Activity of Polysaccharide from Phellinus Fruiting Body

Zhao et al. [104] studied the immunomodulatory effect of polysaccharides from *P. igniarius* fruiting body in the cyclophosphamide-induced immunosuppressive rat model. The functional state of the spleen and thymus can reflect the immune function. The results showed that polysaccharides increased the spleen and thymus index in normal rats and resisted the spleen and thymus atrophy induced by cyclophosphamide.

Toll-like receptor 4 is a pathogen pattern recognition receptor located on the cell membrane of antigen-presenting cells (APC). It can recognize lipopolysaccharide (LPS) and activate innate and acquired immune responses [105,106]. Kim et al. [107] found that polysaccharides from the *P. igniarius* fruit body activate the Toll-like receptor 4 (TLR4) and promote the maturation of dendritic cells. Wang et al. [108] studied the immunomodulatory effect of polysaccharides from the *P. igniarius* fruiting body on HEK-BlueTMhTLR4 cells (a kind of human cells). The authors found that polysaccharides from *P. igniarius* fruit body can be used as TLR4 agonists to activate the TLR and increase the expression of TNF-α, IL-6, IL-12, IL-1β, COX-2, etc. via MyD88 pathway. It also increased the expression of IFN-γ-inducible protein (IP-10) and IFN-β via the TIR domain-containing adaptor inducing interferon-β(TRIF) pathway. Wang et al. [109] reported the activation of TLR4 by polysaccharides from the fruiting body of *P. igniarius* in RAW264.7 cells and peritoneal macrophages. The authors found that *P. igniarius* fruit body polysaccharides improve the proliferation of OVA-specific antibodies and specific spleen cells in mouse serum. Thus, these polysaccharides can be considered safe and effective immune adjuvants. 

Dendritic cells (DC) are specialized antigen-presenting cells. Mature dendritic cells can stimulate initial T cells [110,111]. The expression of IL-12 is a specific marker for activating DC [112,113]. Park et al. [114] found that polysaccharides from fruiting bodies of *P. linteus* can effectively promote the phenotypic and functional maturation of mouse dendritic cells and stimulate DC to secrete IL-12.

### 5.5. Other Pharmacological Activities

Phellinus polysaccharides have been found to promote hematopoiesis and exhibit antiallergic, antidiabetic, and antifatigue effects, as detailed in Table 3. Studies have demonstrated that Phellinus polysaccharides have significant hypoglycemic effects both in vitro and in vivo. For instance, in in vivo experiments using nonobese diabetic mice as a model, Phellinus polysaccharides were able to suppress the development of autoimmune diabetes, reduce blood glucose levels, and decrease pancreatic islet infiltration by regulating cytokine expression. After treatment with Phellinus polysaccharides, the mice showed a mean blood glucose level of 110 mg/dl and delayed splenocyte transfer [115]. Inhibition of α-amylase and α-glucosidase is an effective strategy to control diabetes by reducing glucose absorption. Zhang et al. [116] measured the in vitro hypoglycemic activity of purified polysaccharide from *Phellinus baumii* fruiting bodies and showed high α-glucosidase and α-amylase inhibition activities of 57.37% and 54.10%, respectively. In addition, it also has a strong glucose adsorption capacity, which has a significant inhibitory effect on glucose diffusion.

## 6. Toxicity and Safety

Despite its various pharmacological activities, the toxicity and safety of Phellinus polysaccharides are important considerations. Reports indicate that Phellinus polysaccharides have a very high safety profile. Zhong et al. [86] observed a slight weight gain in Phellinus polysaccharide-treated tumor-bearing mice, with no significant increase in serum ALT and AST levels or lipid peroxide concentrations in the liver and kidney, suggesting that a dose of 200 mg/kg of Phellinus polysaccharides is not toxic to mammals. In recent years, cell chromosome aberration and micronucleus test tests have been used to assess the genotoxicity of drugs. One study found no significant difference (*p* > 0.05) in the chromosomal aberration rate and micronucleus rate of bone marrow cells across various dose groups (2.5 g/kg, 5.0 g/kg, 10.0 g/kg BW) compared to the blank control [126]. In addition, Lin et al. also demonstrated no reproductive toxicity of Phellinus polysaccharides in male mice using mouse sperm aberration and chromosome aberration tests in testicular cells [127].

## 7. Future Prospects

Phellinus polysaccharide has shown many pharmacological effects, including antitumor, immune regulation, hypoglycemic, anti-inflammatory, etc., with very few side effects. Thus, many research studies have been focused on exploring the therapeutic potential of Phellinus in cancer and inflammatory diseases. In recent years, researchers in China and worldwide have been evaluating the pharmacological effects and molecular mechanisms of Phellinus polysaccharides. So far, the research has mainly focused on the fruiting body and mycelium, and very few studies have been carried out on polysaccharides extracted from the culture medium. Moreover, the current research is limited to only in vitro and in vivo experiments. No clinical research was carried out. Although Phellinus polysaccharides have shown extensive pharmacological activities in multiple studies, their exact mechanism of action in vivo, including its absorption, distribution, metabolism, excretion, and so on, is still poorly understood. Extensive further studies are needed to determine their specific targets and mode of action in vivo. Phellinus polysaccharides’ structures are complex and vary depending on extraction and purification techniques. Thus, there are still some challenges in confirming the secondary, tertiary, and quaternary structures of Phellinus polysaccharides. Until now, the main focus was on the primary structures. In addition, studies on pharmacological activities were carried out on crude polysaccharides. There are only very limited studies elucidating the influence of the chemistry of polysaccharides on their pharmacological activities. 

With the enhancement of people’s health awareness and the increase in market demand, the commercial application of Phellinus polysaccharides in medicine, healthcare products, food, and other fields has gradually shown great potential. Japan and South Korea were the first to carry out the cultivation of Phellinus in labs for mass production, and it was used in the manufacture of anticancer drugs and cosmetics [128]. China has great reserves of Phellinus and thus has a greater potential to improve its commercial value. Phellinus polysaccharides can be used in combination with other edible and medicinal mushrooms, such as Ganoderma lucidum and Cordyceps, and complement other health foods. The launch of skin care products with Phellinus polysaccharides as an ingredient is a new direction that can be developed in the future. 

More attention must be paid to improving the culturing techniques for Phellinus, the accurate identification of polysaccharides, and understanding the structure-activity relationship of these polysaccharides. Computer-assisted liquid nuclear magnetic resonance analysis can be utilized to accurately determine the structures of polysaccharides. Additionally, molecular docking and molecular dynamics simulation techniques have been widely employed for studying pharmacological activity and mechanisms [129]. For instance, molecular docking analysis has shown that the mycelium of P. *linteus* has the potential to resist paracetamol-induced liver injury [123]. Wu et al. used molecular dynamics simulation and rigid macromolecule docking, combined with spectroscopy, to elucidate the complex three-dimensional conformation of lentinan [130]. 

Good separation and purification technologies are the keys to improving the commercial and pharmacological application of polysaccharides. We should first concentrate on improving the separation and purification technologies, then technologies to confirm the chemistry of polysaccharides, their pharmacological activities, molecular mechanisms, and structure-activity relationship. In addition, large-scale randomized clinical trials of Phellinus polysaccharides involving a large number of participants, with randomization and double-blind design, are essential to ensure the efficacy and safety of Phellinus polysaccharides in the treatment of various diseases, therefore guiding clinical practice and developing treatment guidelines.

Enhancing separation and purification technologies is crucial for improving the commercial and pharmacological application of polysaccharides. Efforts should first focus on advancing these technologies, followed by refining methods to confirm the chemical properties of polysaccharides, their pharmacological activities, molecular mechanisms, and structure-activity relationships. Conducting large-scale randomized clinical trials on Phellinus polysaccharides is essential to confirm their efficacy and safety in treating various diseases. These trials should involve a significant number of participants and employ randomization and double-blind designs to ensure robust results. This approach will help guide clinical practice and the development of treatment guidelines based on the efficacy and safety of Phellinus polysaccharides.

## Figures and Tables

**Table 1:** The advantages and disadvantages of extraction methods.

Method	Advantages	Disadvantages
Hot water extraction	Simple operation, low equipment requirements	Long extraction time and low efficiency
Ultrasonic-assisted extraction	Low energy consumption, short extraction time, high extraction rate	Influenced by ultrasonic attenuation coefficient
Microwave-assisted extraction	Short heating time, high extraction rate, high selectivity	Active ingredients may be wasted or damaged mechanically
Enzyme-assisted extraction	Mild conditions, no pollution, easy removal of impurities	Highly influenced by costs and enzyme digestion time

**Table 2:** The structural characteristics of Phellinus polysaccharides.

Name	Source of Polysaccharides	Purification Method	Molecular Weight (Da)	Monosaccharide Composition	Monosaccharide Composition Ratio	Types of Main Linkages	Ref.
PIP60-1	fruiting bodies of *P. igniarius*	DEAE-Sepharose Fast-Flow and High-Resolution Sephacryl S-400	1.71 × 10^4^	L-fucose, D-glucose, D-mannose, D-galactose, 3-O-Me-D-galactose	1:1:1:2:1		[38]
*P. igniarius* polysaccharide	fruiting bodies of *P. igniarius*		18,518	glucose, mannose, galactose, xylose, arabinose, rhamnose			[44]
PBF6	fruiting bodies of *P. igniarius*	DEAE-Sepharose Fast-Flow column and Sephacryl S-100 High-Resolution column	3.23 × 10^5^	glucose		→3)-β-D-Glcp-(1→3)-β-D-Glcp-(1→6)-β-D-Glcp-(1→	[41]
*P. linteus* polysaccharide	fruiting bodies of *P. linteus*		20,708	glucose, mannose, galactose, xylose, arabinose, rhamnose			[44]
*P. linteus* polysaccharide	fruiting bodies of *P. linteus*	DEAE-cellulose anion-exchange chromatography, Sepharose CL-4B gel filtration chromatography		mannose, Galactose, glucose, arabinose, xylose		β-(1,3)-D-Glc	[54]
PL-A	fruiting bodies of *P. linteus*	DEAE-cellulose column (4 cm × 60 cm), Sephadex G-50 gel column chromatography (1.6 cm × 40 cm)	14.2 × 10^3^	glucose, mannose		β-(1→3)-Glu	[17]
PL-B	fruiting bodies of *P. linteus*	DEAE-cellulose column (4 cm × 60 cm), Sephadex G-50 gel column chromatography (1.6 cm × 40 cm)	22.2 × 10^3^	glucose, mannose, rhamnose		β-(1→3)-Glu	[17]
P1	fruiting bodies of *P. linteus*		1.88 × 10^4^	L-fructose, D-rhamnose, D-galactose, D-glucose, D-xylose, D-mannose, 3-O-Me-D-galactose	1:3.12:33.51:2.0:4.03:1.09:2.87		[60]
PIP-1	*P. igniarius* mycelia	DEAE-Sepharose Fast-Flow column (2.6 × 40 cm) and a Sepharose CL-4B column eluting		mannose, glucose, galactose	2.41:87.74:3.86		[43]
PIE	*P. igniarius* mycelia	Ethanol precipitation and Sepharose G-100 gel filtration	12 × 10^3^	Xylose, mannose, fucose, glucose, galactose	2.3:1:6.4:22.1: 19.83		[46]
PIPS	*P. igniarius* mycelia		3.1 × 10^3^	glucose, rhamnose, mannose	11.0:14.0:1.0		[45]
IPs	*P. igniarius* mycelia	DEAE-52 cellulose anion-exchange column chromatography	5.7 × 10^3^~6.1 × 10^6^	glucose, mannose, galactose	15:4:1		[61]
PIP1	Mycelia of Liquid-cultured *P. igniarius*	Ethanol precipitation and sepharose CL-6B gel filtration	1.7 × 10^4^	glucose, galactose, mannose	3.70:4.06:1.00	(1→3)-Glc (1→4)-Man	[58]
FIII-1	Mycelia of Liquid-cultured *P. linteus*		10^6^~2 × 10^6^	Fucose, xylose, mannose, glucose, galactose	1.1:3.6:3.4:87.3:4.7		[55]
PPC	*P. linteus* mycelia	DEAE-cellulose column (3 × 45 cm) equilibrated with distilled water	7.3 × 10^3^	D-glucose, D-mannose	3:2		[56]
PLC	*P. linteus* mycelia	DEAE-cellulose and gel-permeation chromatography	1.53 × 10^5^	mannose, galactose, glucose, arabinose, xylose			[57]
PLPS-1	*P. linteus* mycelia	DEAE-52 cellulose and Sephadex G-100 column chromatography	2.5 × 10^5^	glucose, arabinose, fucose, galactose, xylose	21.964:1.336:1.182: 1:1	α-D-Glc(1→4)-α-D-Glc(1→6)	[62]
PLPS-2	*P. linteus* mycelia	DEAE-52 cellulose and Sephadex G-100 column chromatography	2.8 × 10^4^	galactose, glucose, mannose, arabinose, fucose, xylose	14.368:2.594:1.956:1.552:1.466:1	α-(1→3)-D-Glc and α-(1→6)-D-Glc	[62]
*P. linteus* polysaccharide	*P. linteus* fermentation broth		9.4 × 10^3^~15 × 10^3^	glucose, galactose, mannose, arabinose, xylose			[59]
Fr-I	*P. linteus* fermentation broth	SepharoseCL-4B gel filtration chromatography	43.43 × 10^4^	glucose, mannose, galactose			[63]
Fr-II	*P. linteus* fermentation broth	SepharoseCL-4B gel filtration chromatography	3.14 × 10^4^	glucose, mannose, galactose			[47]
Fr-III	*P. linteus* fermentation broth	SepharoseCL-4B gel filtration chromatography	1.29 × 10^4^	glucose, mannose, galactose			[47]
HHM	*P. linteus* crude polysaccharide fermentation broth	Ion-exchange resin, Sephadex G-200, Sephacryl S-400, Sephadex G-100 gel column chromatography	2.84 × 10^6^	glucose			[48]
HLM	*P. linteus* crude polysaccharide fermentation broth	Ion-exchange resin, Sephadex G-200, Sephacryl S-400, Sephadex G-100 gel column chromatography	5.33 × 10^4^	glucose, galactose			[48]
PL-N1	alkaline extract of *P. linteus* mycelia	DEAE-Sephadex A-25	3.43 × 10^8^	arabinose, xylose, glucose, galactose	4.0:6.7:1.3:1.0	(1→4)-linked β-Dxylopyranosyl residues, (1→2)-linked α-D-xylopyranosyl residues, (1→4)-linked α-D-glucopyranosyl residues, (1→5)-linked β-D-arabinofuranosyl residues, (1→4)-linked β-D-xylopyranosyl	[64]
PL-A11	an ammonium oxalate extract of *P. linteus* mycelia	DEAE-Sepharose FF chromatography column (2.6 cm × 40 cm), Sephacryl S-400 HR column (1.5 cm × 60 cm)	1.38 × 10^4^	arabinose, xylose, mannose, glucose	1.1:1.3:1.0:6.6	(1→4)-α-D-glucopyranosyl, (1→2)-α-D-xylopyranosyl, and (1→3)-α-D-arabinofuranosyl	[65]

**Table 3:** Other pharmacological activities of Phellinus polysaccharides.

Pharmacological	Source of Polysaccharides	Experimental Model	Mechanism	Ref.
Promote hematopoiesis	fruiting bodies of *P. igniarius*	immune-injury rats	Increase the number of Hb, globulin, and peripheral white blood cells in normal rat blood; Significant recovery of leukocyte count and hemoglobin content in peripheral blood of immunosuppressed rats	[104]
Anti-allergy	fruiting bodies of *P. linteus*	rat peritoneal mast cell	Inhibit mast cell degranulation and inhibits histamine, TNF-α, IL-6, and other factors related to type I allergy	[117]
Antifatigue	mycelial of *P. linteus*	KM mice	Reduce serum urea nitrogen content and blood lactate content in mice after strenuous exercise	[118]
Antifatigue	Fermentation Broth of *P. igniarius*	KM mice	Reduce blood lactate levels and blood creatine kinase levels	[119]
Antidiabetic nephropathy	*P. igniarius*	diabetic mice	Regulate MMP-2/TIMP-2 balance and inhibit activation of P311/TGF-β1/Snail1 signaling pathway	[120]
Anti-hyperlipemia	mycelia and extracellular of *P. linteus*	hyperlipemia mice	Reduce serum triglyceride (TG), total cholesterol (TC), and low-density lipoprotein (LDL) levels and increase high-density lipoprotein (HDL) levels in hyperlipidemic mice	[121]
Anti-septic shock	*P. linteus*	Septic Shock Mice	Reduce splenocyte production of IL-2, IFN-γ, and TNF-α and enhance spontaneous apoptosis of macrophages and lymphocytes	[122]
Anti-paracetamol-induced liver injury	mycelial of *P. linteus*	Paracetamol-induced liver injury in mice	Reduce cytochrome P450 2E1 (CYP2E1) expression and hepatic cytokine release, therefore increasing the level of phase II enzymes	[123]
Antidiabetic	mycelial of *P. linteus*	type 2 diabetic rats	Play a prebiotic effect, improve gut microbiota, and reduce HFD-induced insulin resistance	[124]
Hypoglycemic	*P. linteus*	HFD fed mice	Restore vitamin B12 level; Rescue hepatic PC/PE ratio and activate FOXO signaling pathway to improve insulin resistance	[125]
